# Buffalo Academy of Medicine

**Published:** 1903-02

**Authors:** N. L. Burnham


### SOCIETY PROCEEDINGS.

### Buffalo Academy of Medicine.

*Section on Medicine, December 9, 1902.*

REPORTED BY N. L. BURNHAM, M. D., Secretary.

The regular meeting of the Medical Section of the Buffalo Academy of Medicine was held in the Academy rooms, Public Library Building, on Tuesday evening, December 9, 1902, the chairman, Dr. ALBERT E. WOEHNERT, presiding. The meeting was called to order at 8.50 p.m. The minutes of the previous meeting were read and approved.

The first paper of the evening, entitled,

PULSATIONS OF THE THORACIC WALL, WITH REPORT OF A CASE OF PULSATING HEMOTHORAX.

was read by Dr. JOSEPH SAILER, of Philadelphia.

Pulsations in the walls of the thorax may be divided into those forms due to excessive action of the heart or bloodvessels, either as a result of disease or nervous influence, and those forms due to some alteration in the pleural contents. Of the latter we can recognise the endo- and eso-pleural pulsations; the latter may be circumscribed, or a diffuse wave or a diffuse expansion. It has been noted in a variety of conditions, of which the most important are empyema of necessity, pyopneumothorax, simple empyema, serous effusion, abscess of the thoracic wall and hemothorax. The great majority of cases occur upon the left side and apparently the most important etiological factor is tension of the mediastinal tissues. This tension is favored by obliteration of the pericardial cavity, by pericardial adhesions, or by pericardial effusion.

In a case of hemothorax, due to rupture of an aneurism of the transverse and descending arches of the aorta, pulsation was observed over the situation of the aneurism, and, in addition, around the base of the chest from the sternum to the vertebral column. This pulsation was distinctly expansible in type. At the autopsy, obliteration of the pericardial cavity was found; the mediastinal tissues were tense and two rents were found in the wall of the aneurism. It is probable that in this case the tension of the mediastinum, the pulsation of the heart and the aneurism, and the pumping of blood through the opening in the aneurism into the pleural cavity, all contributed to the production of pulsation.

Dr. Sailer also reported a case of

EVENTRATION OF THE DIAPHRAGM.

A case of epilepsy developed typhoid fever, and when transferred to the medical ward a tympanitic note was heard over the left side of the chest below the third rib. The heart was displaced to the right, and when the patient swallowed, gurgling was heard over the tympanitic area. The upper border of this tympanitic area moved on respiration. In diagnosticating the case there was considered: high position of the diaphragm, pneumothorax, cavity in the lung and diaphragmatic hernia. Careful consideration of the physical signs led to the preference being given to high position of the diaphragm. At the autopsy this was found to be the case, apparently due to an extreme degree of hypoplasia of the left lung, which was further deformed by being divided into four lobes. This condition is known as eventration of the diaphragm, a name given by Courveilhier in the early part of the 19th century. Since his original report, thirteen cases, not including the present one, have been reported, all characterised by hypoplasia of the left lung, dislocation of the heart to the right, and high position of the diaphragm.

In another series of cases in which hypoplasia of the left lung has existed, there has been dislocation of the heart to the left and backward and the diaphragm has not been markedly displaced. It seems more reasonable, in view of the fact that the primary condition is the same in both series, to group the two conditions together and speak of them as hypoplasias of the left lung with dextrocardia and high position of the diaphragm, or with dislocation of the heart backward and to the left.

Dr. Sailer exhibited some very excellent skiagraphic work of these two cases. The plates showed such minor detail as coils of intestine, area of congested lung, height of diaphragm, and the like.

DISCUSSION.

Dr. Rochester congratulated the essayist upon the very excellent clinical work done upon these cases and also the high grade of *x*-ray work which was being done at the Philadelphia Hospital. He had seen a case of pulsating chest in a patient suffering with empyema and a hypertrophied heart. The pulsation occurred in the axillary region on the left side. Autopsy revealed an adhesion between the pleural in the region of the heart. This adhesion assisted pulsation. Dr. Rochester moved a vote of thanks to the reader of the papers.

The motion was seconded by Dr. Charles G. Stockton, who considered Dr. Sailer's classification a sensible one. Dr. Stockton had seen a case of pulsating hemothorax primarily with the pulsation soon disappearing. The autopsy revealed the left thorax filled with blood and a ruptured aorta.

Dr. Allen A. Jones referred to gastric stagnation as a symptom in diaphragmatic hernia. He requested Dr. Sailer to explain the cause of pulsating pneumothorax.

The paper was also discussed by Dr. Lyon. Dr. Sailer, in closing the discussion, said, among other things, that air in the thorax must be under higher tension than the external air to produce pulsating pneumothorax.

Dr. Charles S. Jones read a paper entitled, Nephritis in Infants. He reported four cases of nephritis in infants which he had studied in his practice and suggested frequent careful urinalysis in children.

The paper was discussed by Dr. Dewitt H. Sherman and Dr. Allen A. Jones.

Meeting adjourned at 11 p. m. Number of Fellows present, 40.

---

### *Section on Pathology, December 16, 1902.*

REPORTED BY JACOB S. OTTO, M. D., Secretary.

The regular monthly meeting of the Section of Pathology of the Buffalo Academy of Medicine, was held in the Academy rooms, Buffalo Library Building, December 16, 1902, the chairman, Dr. I. P. LYON, presiding.

Dr. CHARLES WARDELL STILES, Ph. D., Zoologist of the United States Public Health and Marine Hospital Service, at Washington, D. C., gave an interesting and instructive talk on

### THE PREVALENCE AND GEOGRAPHIC DISTRIBUTION OF HOOKWORM DISEASE (UNCINARIASIS) IN THE UNITED STATES, making special reference to a new species of hookworm (Uncinaria Americana) which was named by him and reported in *American Medicine*, May 10, 1902, p. 777. Dr. Stiles is entitled to the credit of differentiating this species of hookworm, which is very prevalent in the Southern States and which is most important in that it leads to such severe pathological conditions. The following abstract of his remarks is presented:

*Uncinaria Americana* (new-world hookworm) is differentiated from *U. duodenalis* (old-world hookworm) and is seen to approach *U. stenocephala* of dogs in some characters and *U. cerua* of sheep in others.

First establishing the occurrence of *U. Americana* in the three widely separated points, Texas, Virginia and Porto Rico, he obtained authorisation from Surgeon-General Wyman to make investigation within this triangular territory. He commenced his investigations in Virginia in the fall of this year (1902) and worked directly southward from Washington, visiting orphan asylums, schools, mines, plantations, cotton mills, and the like, from which abundant material was obtained, not, however, without difficulty in some places. It was soon found that the disease arose almost exclusively on sandy soil and in rural districts, practically never in towns, whereas malaria, with which it had been confounded, is found on clay soil. It was also found to be more prevalent among the whites than among the negroes.

To Dr. Stiles, credit is due for a full description of the symptoms of this disease, which in a marked case are well defined. A person suffering with a severe case of uncinariasis presents first the symptoms of a marked anemia. The lips are blanched, secondary pigmentation of the skin occurs, the skin becoming parchment-like and of a yellowish-brown hue. There is extreme emaciation, while the abdomen becomes protuberant (pot belly). A special symptom of the eye was noted, which has never been previously described, the pupils becoming markedly dilated when the patient's eyes are fixed on any object. After this symptom was discovered it was noted in every case of the disease whereas, by contrast, it was found in only two persons (sons of an inebriate) not affected with uncinariasis.

There are also present palpitation of the heart, shortness of breath, visible pulsation of the vessels of the neck and swelling of the feet and abdomen. These symptoms are so constant that the patients, when asked with what they are afflicted, frequently reply "heart disease and swollen feet." The pot belly is caused by ascites and tympanites, and sometimes hepatic and splenic enlargement. The disease in its extreme form generally causes a perversion of the appetite, of one form or another, and persons were observed to eat such things as mud, sand, clay, tobacco, salt, salt and lemons, rags, chips of wood, and even live mice. This feature of the disease has also been noted in animals. Eighteen per cent. of the seals of Alaska die from uncinariasis, and stones are found in the stomach post-mortem.

If the disease begins in childhood, at which period are shown the most marked symptoms, the growth, both mental and physical, is stunted, so that an adult may appear to be ten or twelve years of age. This feature has a direct and important bearing on the question of child-labor in the South, and Dr. Stiles took occasion to defend the Southern millowners who have been more or less severely criticised for the physical and mental condition of the children employed in the mills. The greater number of them come from the sandy farming areas and are found to have been suffering from uncinariasis before they entered the mills, and from conversation with the children thus employed it was learned that they were healthier in the mills than they were in their homes.

The clinical determination of a case of uncinariasis depends on the above symptomatology plus certain specific tests.

Test 1.—The detection of ova in the feces by means of the microscope. The adult parasites are not found, except after anthelmintic treatment.

Test 2.—In the absence of the microscopical test a small mass of feces may be placed on filter paper and after twenty minutes removed. In 80 per cent. of cases of this disease a marked reddish-brown stain is left on the paper, which is due to the presence of blood in the feces. The hemorrhage is caused by a piercing of the mucous membrane by the parasite.

The examination of the blood shows marked loss of red corpuscles and hemoglobin, a variable leucocytosis and a marked eosinophilia. As eosinophelia may occur with other intestinal parasites, it is merely suggestive in this disease.

*Treatment.*—Thymol is practically a specific, and should be given in large doses, 2 grammes at 8 o'clock a.m. and 10 o'clock a.m., followed at 12 o'clock noon by a brisk saline cathartic. It is usually necessary to repeat the medication at the end of a week. Thereafter, weekly examinations of the stools for two or three weeks should be made to determine the effectiveness of the treatment. Frequently a further course of treatment is found necessary. Care must be taken not to administer thymol in alcoholic solution or at the same time with alcohol, as toxicity and convulsions may result.

### DISCUSSION.

Dr. Allen A. Jones inquired about the type of anemia that was associated with the disease.

Dr. A. L. Benedict wished to know how many slides were necessary to detect the parasite.

Dr. C. C. Frederick asked if infection came from water supply or by inhalation.

Dr. Julius Ullman asked if the hemorrhages were severe enough to account for the anemia.

Captain Riley, Assistant Surgeon U. S. Army, gave an interesting account of his experience with uncinariasis in the Philippines, where he had seen many cases among the natives and soldiers. He believed that *uncinaria Americana* causes a more severe infection than the European type, which latter is the species found in the Philippines. He has seen some cases in which the ovum was present without symptoms other than those of slight indigestion. Natives use the emulsion of turpentine in the treatment of the diseases. It is necessary to use caution in the administration of thymol, as the natives and soldiers drink vinol, an alcoholic beverage.

Dr. H. U. Williams asked if there was any other factor than hemorrhage in producing the anemia.

In closing the discussion Dr. Stiles stated that the anemia was of the pernicious type and the disease was often diagnosticated pernicious anemia. He considered the blood examination an indirect method of diagnosis, as an eosinophilia would merely suggest infection by an intestinal parasite and would direct one to an examination of the feces.

The anemia is produced in the following way: the parasite attaches itself to the intestinal wall and sucks blood for a time, then lets go and travels to another part of the intestine, leaving behind small bleeding points, causing the hemorrhage. This process leads to thickening of the intestine and a consequent interference with absorption of nutritive materials. Dr. Stiles believed there was also produced by the parasite a toxin which is a factor in causing the anemia and especially the eye symptoms. Uncinariasis is a filth-disease and the infection is directly from the dirt on the hands, not through inhalation.

One slide preparation usually suffices to reveal the eggs. The adult is rarely found, except after anthelmintic treatment, when many hundreds of the parasites may be found.

Dr. Lyon stated that the discovery announced tonight by Dr. Stiles before our Academy was of immense importance in its humanitarian aspect. It was no mere discovery of a new variety of parasite, but it meant the recognition for the first time in the United States of the existence of a widespread affection, afflicting untold thousands of our citizens, and condemning them to a life of more or less continued suffering. The recognition of the disease means its control and its ultimate stamping out. This discovery ranks in its importance to humanity with the two other great medical discoveries of recent years,—the discoveries of the relation of mosquitoes to malaria and to yellow fever.

A vote of thanks was moved, seconded and carried, expressing the appreciation of the section of Dr. Stiles's interesting and instructive address.

Members present, 46.

